# Female Genital Mutilation in Saudi Arabia: A Systematic Review

**DOI:** 10.7759/cureus.19300

**Published:** 2021-11-06

**Authors:** Hashim H Almeer, Ali A Almulla, Abdulelah A Almugahwi, Mohamad Z Alzaher, Mustafa M Alshammasi, Ritesh G Menezes

**Affiliations:** 1 Internal Medicine, Imam Abdulrahman Bin Faisal University, Dammam, SAU; 2 Pathology, Imam Abdulrahman Bin Faisal University, Dammam, SAU

**Keywords:** saudi arabia, trauma, female circumcision, female genital cutting, female genital mutilation

## Abstract

Female genital mutilation (FGM) or circumcision refers to the unnecessary procedure that damages or removes the external genitalia of females. It is mostly practiced in African countries and some Asian regions, particularly the Middle East, and is performed because of cultural, religious, and social reasons. FGM can negatively affect the lives of women and lead to devastating consequences, ranging from immediate to long-term complications. These complications can be in the physical, psychological, reproductive, or sexual health domains. In this systematic review, we aim to highlight the prevalence and practice of FGM in Saudi Arabia. We conducted a literature search at PubMed to identify studies related to the practice of FGM reported from Saudi Arabia. The results indicate that FGM can still be found in Saudi Arabia both in Saudi women and non-Saudi residents. Most of the non-Saudi women with FGM were Sudanese, Somali, Eritrean, and Egyptian. FGM is prevalent in regions such as Jeddah and Hali, Al Qunfudhah Governorate, Saudi Arabia. FGM is considered illegal in most countries around the world. However, in Saudi Arabia, there is no clear and specific law against the practice of FGM. More research on the practice of FGM in Saudi Arabia needs to be conducted to get a better grasp of the true nature of the problem in the country, which could potentially lead to specific and clear legislation that would prevent the social evil of FGM in Saudi Arabia.

## Introduction and background

Female genital mutilation (FGM) is any procedure that involves the partial or total removal of external female genitalia for nonmedical purposes [1]. This practice is frequently done by traditional circumcisers who have some role in the communities, such as birth attendants. In many areas, healthcare providers perform mutilation due to the belief that the procedure is safer when medicalized [1]. The reasons why FGM is performed differ between countries and time periods. Also, it is a mix of historical, social, and cultural factors within communities and families [1].

The World Health Organization (WHO) has classified FGM into four major types (Table 1) [1]. The WHO estimated the costs of treating complications of FGM to be 1.4 billion US dollars during a one-year period in 27 countries in 2018 [2]. The cost is expected to be 2.3 billion in the next 30 years if the prevalence of FGM procedures remains the same [2].

**Table 1:** WHO classification of female genital mutilation [1]

Type	Definition
I	"Total or partial removal of the clitoral glans and/or the fold of the surrounding skin (prepuce)"
II	"Total or partial removal of clitoral glans and the labia minora, sometimes with that of the labia majora"
III	"Narrowing of the vaginal orifice, sometimes with infibulation (excision of the clitoris)"
IV	"Includes all other nonmedical, harmful procedures of the external female genitalia"

The practice is mainly performed in multiple regions of Africa; some countries in Asia, particularly the Middle East; and among migrants from these regions [2]. It is estimated that more than 200 million women alive today have undergone FGM [2]. In addition, approximately three million females are at risk of FGM, the majority of whom are less than 15 years of age [3]. In this systematic review, we aim to highlight the prevalence and practice of FGM in Saudi Arabia.

## Review

Methods for literature search

A literature search was conducted using the PubMed database on October 1, 2021. The terms included in the search were "female genital mutilation," "FGM," and "female circumcision." In addition, to limit the search to Saudi Arabia, the terms "Saudi Arabia," "Kingdom of Saudi Arabia," and "KSA" were used (Table 2). The inclusion criteria consisted of studies related to FGM reported from Saudi Arabia and published in English. There were no year restrictions considered during the search.

**Table 2:** Search strategy employed at PubMed

Database	PubMed
Search terms	(female genital mutilation OR FGM OR female circumcision) AND (Saudi Arabia OR KSA OR Kingdom of Saudi Arabia)
Search details	("circumcision, female"[MeSH Terms] OR ("circumcision"[All Fields] AND "female"[All Fields]) OR "female circumcision"[All Fields] OR ("female"[All Fields] AND "genital"[All Fields] AND "mutilation"[All Fields]) OR "female genital mutilation"[All Fields] OR "FGM"[All Fields] OR ("circumcision, female"[MeSH Terms] OR ("circumcision"[All Fields] AND "female"[All Fields]) OR "female circumcision"[All Fields] OR ("female"[All Fields] AND "circumcision"[All Fields]))) AND ("saudi arabia"[MeSH Terms] OR ("saudi"[All Fields] AND "arabia"[All Fields]) OR "saudi arabia"[All Fields] OR "KSA"[All Fields] OR ("saudi arabia"[MeSH Terms] OR ("saudi"[All Fields] AND "arabia"[All Fields]) OR "saudi arabia"[All Fields] OR ("kingdom"[All Fields] AND "saudi"[All Fields] AND "arabia"[All Fields]) OR "kingdom of saudi arabia"[All Fields]))
Search translations	*female genital mutilation:* "circumcision, female"[MeSH Terms] OR ("circumcision"[All Fields] AND "female"[All Fields]) OR "female circumcision"[All Fields] OR ("female"[All Fields] AND "genital"[All Fields] AND "mutilation"[All Fields]) OR "female genital mutilation"[All Fields] *female circumcision:* "circumcision, female"[MeSH Terms] OR ("circumcision"[All Fields] AND "female"[All Fields]) OR "female circumcision"[All Fields] OR ("female"[All Fields] AND "circumcision"[All Fields]) *Saudi Arabia:* "saudi arabia"[MeSH Terms] OR ("saudi"[All Fields] AND "arabia"[All Fields]) OR "saudi arabia"[All Fields] *Kingdom of Saudi Arabia:* "saudi arabia"[MeSH Terms] OR ("saudi"[All Fields] AND "arabia"[All Fields]) OR "saudi arabia"[All Fields] OR ("kingdom"[All Fields] AND "saudi"[All Fields] AND "arabia"[All Fields]) OR "kingdom of saudi arabia"[All Fields]
Sorted by	Best match
Search results (items/articles) obtained	34
Items that met the inclusion criteria to be included in the present literature review	13

Results of literature search

**Figure 1:**
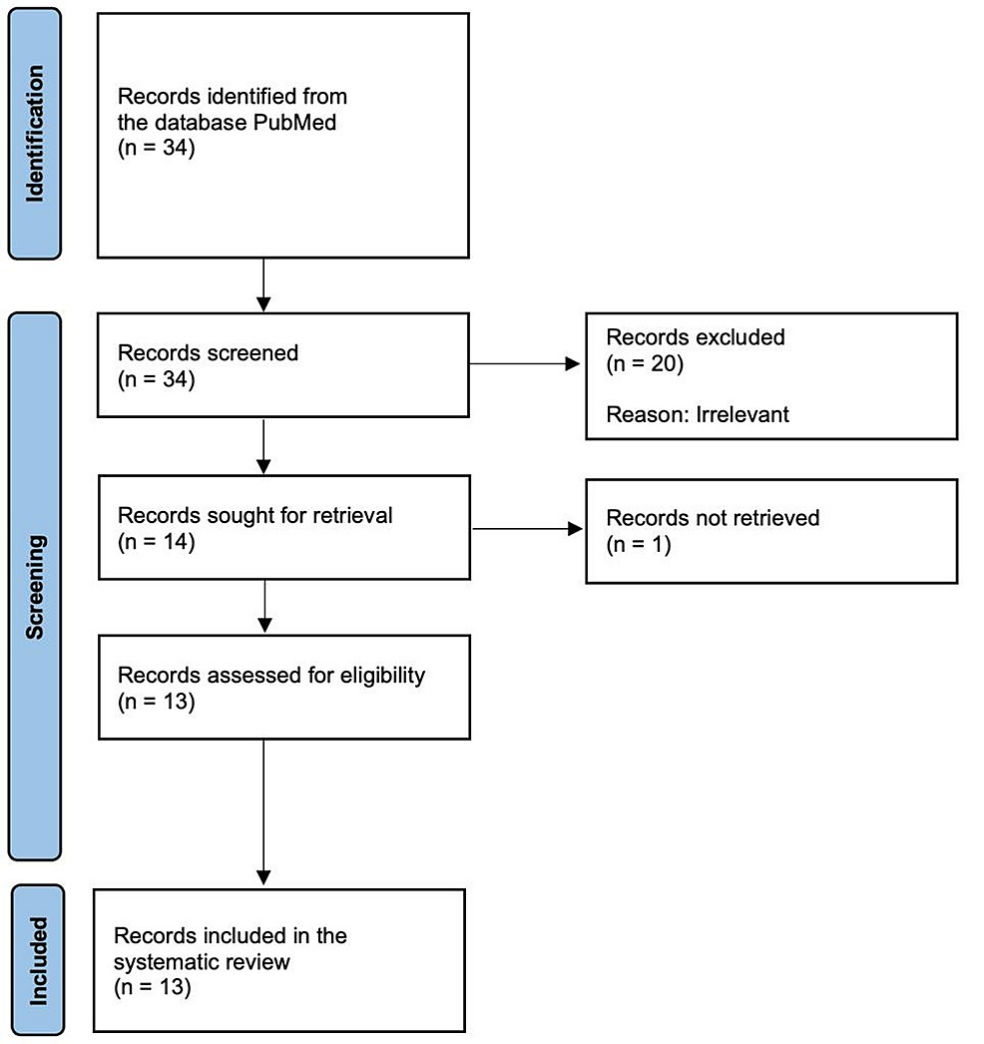
Study selection for review (PRISMA flowchart; http://www.prisma-statement.org/)

The PRISMA flowchart (Figure 1) was followed to identify relevant articles. A total of 34 items/articles (records) were identified using the search terms, and 13 retrievable articles [4-16] that met the inclusion criteria were included in the systematic review.

Table 3 depicts the incidence and safety of defibulation in FGM patients. The prevalence and complications of FGM in Saudi Arabia are represented in Table 4 and Table 5, respectively.

**Table 3:** Summary of the reviewed articles (defibulation during vaginal delivery in women with FGM in Saudi Arabia)

Reference	Setting	Study Design	Sample Size	Important Results
Rouzi et al. [6]	King Abdulaziz University Hospital, Jeddah, Saudi Arabia	Retrospective hospital-based study	631	Out of a total of 631 pregnant women, 27% had type III FGM and underwent delivery with defibulation, and 73% did not have FGM and underwent delivery without defibulation. Regarding the outcomes, there were no statistically significant differences between the two groups in the onset of labor, duration of the stage of labor, blood transfusions, and the duration of maternal hospital stay. However, instrumental deliveries and blood loss were more common in women who underwent delivery without defibulation. This study concluded that defibulation during delivery in women with type III FGM was a safe practice and it did reduce the risk of obstetric complications.
Rouzi et al. [11]	King Abdulaziz University Hospital, Jeddah, Saudi Arabia	Case–control study	388	There were no statistically significant differences between women (n = 388) with type III FGM who underwent defibulation during vaginal delivery and those who did not (n = 388; control group) in the duration of labor, episiotomy rates, and blood loss.
Rouzi et al. [15]	King Abdulaziz University Hospital, Jeddah, Saudi Arabia	Retrospective hospital-based study	325	Among the 325 patients, 158 patients (48.6%) had infibulation (FGM) and needed defibulation to have a normal vaginal delivery. In contrast, 116 patients (35.7%) who did not have FGM had a normal vaginal delivery without defibulation, and the remaining 51 patients (15.7%) who did not have FGM delivered by Cesarean section. There were no statistically significant differences between the two groups regarding the duration of labor, rates of episiotomy and vaginal laceration, blood loss, and the duration of maternal stay in hospital.

**Table 4:** Summary of the reviewed articles (prevalence of FGM in Saudi Arabia)

Reference	Setting	Study Design	Sample Size	Important Results
Rouzi et al. [5,7]	King Abdulaziz University Hospital, Jeddah	Cross-sectional survey	963	Of the participants, 18.2% (n = 175) self-reported that they had FGM. Of these, 110 were Saudis and 65 non-Saudis. More than 50% of the non-Saudis were Yemenis. The rest of the non-Saudis were from African countries, including Sudan, Egypt, Somalia, and Ethiopia. About 25% of the women with FGM were unmarried, and the rest of the cohort was formed by married, divorced, or widowed women. About 42% of the women with FGM had a low monthly income of less than 5000 Riyals. The age at which FGM was carried out was within one week after birth in more than 50% of the women (n = 101) and at the age of 6.9 ± 0.1 years (mean ± SD) in about 25% (n = 42) of the women; 18.3% (n = 32) were not aware of the age at which FGM was carried out. The procedure was carried out by a traditional birth attendant/midwife, physician/nurse, or relative in 37.1%, 21.7%, and 20% of the women, respectively. The victim’s house (56.6%) was the most frequent place where FGM was carried out. This place was a hospital/private clinic or midwife’s house in 23.4% and 4.6% of cases, respectively.
Milaat et al. [8]	Houses located in Hali, Al Qunfudhah	Cross-sectional survey	218	Of the participants, 80.3% (n = 175) self-reported that they had FGM. The age at which FGM was carried out was at seven years or less in 59.4% of cases and 18 years in 35.4% of cases. The procedure was mainly performed by doctors or nurses in 91.4% and 5.7% of cases, respectively. Those whose parents had a university degree or higher and those with better family income showed lower FGM rates. In contrast, the rates of FGM were higher among those with consanguineous parents and those whose mothers were married at a younger age.
Rouzi et al. [9]	Doctor Erfan and Bagedo Hospital, Jeddah	Cross-sectional, observational, prospective study	107	The study participants were Sudanese women with 39%, 36%, and 25% having had type I FGM, type III FGM, and type II FGM, respectively.
Alsibiani et al. [13]	King Abdulaziz University Hospital, Jeddah	Case–control study	130	Of the participants, 40.8% (n = 53) had type I or type II FGM, 42.3% (n = 55) had type III FGM, and the type of FGM was unclear in 16.9% (n =22).
De Silva et al. [16]	King Abdulaziz University Teaching Hospital, Jeddah	Case–control study	173	Of the 173 patients with FGM who underwent delivery, 167 were Sudanese, representing 96.5% of the women with FGM. The rest of the women with FGM were Somali, Eritrean, and Egyptian. There were no Saudis with FGM.

**Table 5:** Summary of the reviewed articles (complications of FGM in Saudi Arabia)

Reference	Setting	Study Design	Sample Size	Important Results
Rouzi et al. [9]	Doctor Erfan and Bagedo Hospital, Jeddah	Cross-sectional, observational, prospective study	107	A substantial proportion of women with FGM experienced sexual dysfunction. The anatomical extent of FGM was related to the severity of sexual dysfunction.
Rouzi et al. [10]	King Abdulaziz University Hospital, Jeddah	Case report	1	Type I FGM resulted in the complete closure of the vagina.
Rouzi et al. [12]	King Abdulaziz University Hospital, Jeddah	Retrospective, hospital-based study	32	Of the 32 patients, who underwent surgery for epidermal clitoral inclusion cysts, 15 had a history of FGM, thus indicating that clitoral cysts are not an uncommon complication of FGM.
Alsibiani et al. [13]	King Abdulaziz University Hospital, Jeddah	Case–control study	130	The association between type I and type II FGM with sexual dysfunction was found to be statistically significant.
De Silva et al. [16]	King Abdulaziz University Teaching Hospital, Jeddah	Case–control study	173	Candida albicans infection, mixed genitourinary tract infections, and E. coli bacteriuria were found to be in significantly higher proportions during pregnancy in women with FGM. The significant labor complications in women with FGM included prolonged stage II of labor, postpartum hemorrhage, and urethral tears.
Rouzi et al. [14]	King Fahad Armed Forces Hospital, Jeddah	Retrospective, hospital-based study	21	All patients included in this case series had epidermal clitoral inclusion cyst as a long-term complication of type I FGM.

Discussion

History of Female Genital Mutilation

FGM or circumcision is an old procedure that has been practiced for centuries [5]. Multiple factors play a role in the practice of FGM, including cultural, religious, and social factors [5]. The origin of FGM is unclear. Nevertheless, it is proposed that the practice originated in ancient Egypt, as mummies from the fifth century were found to be circumcised [17]. The origin of FGM is also theorized to be slave trading, as people moved from the western regions of Africa and the western shore of the Red sea [17]. Furthermore, an extension from the Middle East to Africa by the Arab traders has also been theorized. Despite the controversy of its origin, FGM is considered a common practice worldwide [2].

The WHO stated that more than 200 million women have been affected by FGM in 2017 [2]. It is commonly practiced in the northern, eastern, and western regions of Africa [17]. In addition, several Middle East countries such as Iraq and Yemen have also been considered as common regions of FGM [6]. In Saudi Arabia, there was a belief that FGM is not common due to presumed governmental restrictions and religious prohibition [5]. However, recent studies have shown that the practice of FGM exists in Saudi Arabia [5-9]. Moreover, there are some quotes about FGM reported in the Islamic literature by the Prophet Mohammad (*peace be upon him*) [18]. The most narrated quote was "*A woman used to perform circumcision in Medina. The Prophet said to her: Do not cut severely as that is better for a woman and more desirable for a husband*" [18]. In the pre-Islamic era, there was occasionally a practice of partial or total removal of the clitoris. The reason for this practice in that era is unclear; however, relieving the discomfort of riding the horses or camels is a possible reason [18].

Effects and Complications of Female Genital Mutilation

FGM may have various consequences, some immediate and some arising later in life. These consequences can be related to physical, psychological, reproductive, and sexual health [19-21]. A questionnaire-based cross-sectional study conducted in Saudi Arabia reported that over two-thirds of the study participants were aware of the health-related complications of FGM [4]. The occurrence and severity of the adverse events due to FGM are dependent upon many factors, including the hygiene of the environment, the skills of the person performing the procedure, the resistance of the child, and how susceptible the victim is to infections [21]. FGM has been previously nicknamed "three feminine sorrows" [22]. This refers to the pain and suffering that the victim of FGM experiences during three points of her life: immediately after the procedure, during sexual intercourse, and while giving birth [22].

The immediate consequences of FGM include hemorrhage and extreme pain that may lead to shock and loss of consciousness and even death [21]. Women who undergo FGM usually have their legs bound for a period after the procedure, leading to the restriction of fluid drainage and thus increasing the chances of infection of the female reproductive system and the urinary tract, as well as leading to chronic kidney disease and poor wound healing [21]. Furthermore, damage to adjacent tissues and structures such as the urethra is not uncommon, and the damage to these structures may lead to long-term consequences such as urinary retention, recurrent urinary tract infections, and epidermal inclusion cysts [9,12,21]. Epidermal inclusion cyst is not a rare complication of FGM [12]. A study revealed that 21 patients who underwent type I FGM presented with a mass in the vulva that was diagnosed as an epidermal clitoral cyst secondary to type I FGM [14]. Another complication secondary to FGM is a case of a completely closed vagina. It was reported from King Abdulaziz University Hospital, Jeddah. The patient was a 16-year-old Eritrean who complained of urinary retention that was associated with a history of recurrent urinary tract infections [10].

Sexual dysfunction is commonly associated with FGM, and the degree of the dysfunction increases with the severity and extent of the mutilation [9]. However, a study published in 2010 showed that even type I and type II FGMs are associated with sexual dysfunction [13]. The sexual dysfunction arising from FGM is found to be across all sexual domains such as libido, arousal, orgasm, satisfaction, lubrication, and pain [9]. The clitoris is believed to be the most important organ in female sexual health, and most FGM procedures involve the mutilation of this critical organ [1,23]. Obstetric complications frequently occur in patients with FGM, and these complications include postpartum hemorrhage, genital tract trauma, and perineal tearing [24]. FGM is also associated with increased rates of Cesarean section and extended maternal hospital stay [24,25]. In addition, a study revealed an increased incidence of vaginal candidiasis in circumcised women compared with the control group [16]. However, a study based in Australia showed that females with FGM had similar obstetric outcomes to females without FGM, except that the FGM group had a higher risk of Cesarean section and first- and second-degree perineal tears [26]. This might indicate that the hospital facilities and expertise of the staff have an influence on the outcomes of delivery in women with FGM. Furthermore, a procedure in which the small vaginal opening is cut open, known as defibulation, is required for patients with type III FGM to facilitate an unobstructed birth [11,15]. A recent study published in 2020 suggested that defibulation reduces the incidence of obstetric complications in women with type III FGM [6].

The procedure of FGM can be very traumatizing, especially if done at a young age. Posttraumatic stress disorder, depression, and anxiety have been frequently documented to be associated with FGM [27-29]. The feeling of being betrayed, wanting to cry, and being lonely along with sleep disturbances and headaches were also frequently reported [29]. However, more studies need to be conducted to study other associated factors such as physical and sexual violence.

Female Genital Mutilation in Saudi Arabia

The practice of FGM for reasons that are not related to medical treatment has been widely spread over the years, affecting females particularly in the region of Africa and the Middle East [9]. In a study performed at King Abdulaziz University Hospital in Jeddah [7], it was concluded that FGM is being practiced among both Saudi and non-Saudi women residing in Jeddah. The study included 963 women between the ages of 18 and 75 years from Saudi and other countries, mainly Yemen, Sudan, Egypt, Somalia, and Ethiopia, with the majority of them being Muslims (79.1%). The study showed that 18.2% of the participants had FGM. Those who had FGM were older and married and had lower monthly income compared with those who did not undergo FGM. Moreover, FGM practice was less common among Saudi women compared with the other nationalities residing in Saudi Arabia. Furthermore, the study indicated that the nationality and the age at which FGM took place had an association as women with Saudi nationality performed FGM at an earlier age compared with women from other nationalities [7].

Another study conducted in Hali, Al Qunfudhah Governorate, Saudi Arabia, was based on a community household survey to detect the prevalence of FGM in females who are ≤18 years old [8]. The study concluded that FGM is still rooted in the culture of people in rural and semi-urban areas. Based on the Hali official map, the region was divided into 30 clusters. The survey was directed to 12 houses in each cluster and covered 218 females. The results showed that the majority of females had their circumcision performed at seven years of age or less (59.4%). Female circumcision was mostly done by doctors (91.4%). Circumcision was less common in females whose parents had better education and good income and were unrelated and whose mothers got married at an older age. However, the differences were statistically insignificant [8].

Forensic and Legal Aspects of Female Genital Mutilation

Given that FGM has no real benefit and can lead to devastating complications both in the short term and long term as discussed earlier, it is not surprising that it is illegal in most countries around the world. The United States, for example, and the United Nations consider FGM to be a crime and a violation of human rights. People who are charged with the offense of committing FGM in these countries can face serious consequences and might serve time in prison [30]. Furthermore, even in countries where it is technically not illegal, it is heavily frowned upon and constantly faces criticism from the media. Multiple protests and calls for action are continuously being carried out to ban the evil practice of FGM across the world. For instance, the “28 Too Many” is a charity organization based in England and Wales that aims to help implement and enforce laws against the practice of FGM in 28 African countries [31].

The practice of FGM in Saudi Arabia is controversial yet rarely addressed. Some claim that it only exists in some parts of the southern regions of the country, and others claim that it is present in all regions. Part of the controversy stems from the fact that there is no clear and specific law against FGM in Saudi Arabia. However, hospitals and clinics across the country do not perform FGM, at least not officially. On the other hand, most victims of FGM report that the procedure was done by medical professionals [7,8]. The issue of the law being unclear regarding the practice of FGM is not limited to Saudi Arabia since neighboring countries such as the United Arab Emirates also share this predicament [32].

Further nationwide research regarding FGM needs to be conducted in Saudi Arabia to determine the prevalence and actual impact of FGM at the national and subnational levels, as well as to determine whether this practice is different or not, compared with other countries where FGM is practiced. In addition, more research is needed to focus on the social perspective of FGM and whether there is a social norm regarding FGM that affects the decision of the victims of FGM to undergo such practice in Saudis and non-Saudis residing in Saudi Arabia. Furthermore, the information gained from such studies would provide guidance to decision-makers about the nationwide prevalence of FGM and, most importantly, to construct a legal change to enact clear and specific laws against the practice of FGM in Saudi Arabia. 

## Conclusions

FGM has been in practice for centuries in many countries around the world. It is associated with multiple serious complications that in severe cases might even lead to death. The United Nations considers FGM as a violation of human rights, as it might lead to various physical, psychological, and sexual adversities.

Although FGM is illegal in many countries, there are no clear and specific legal restrictions regarding the practice of FGM in Saudi Arabia. It was thought that FGM is not common in Saudi Arabia; however, recent studies show that it is prevalent in Jeddah and Hali in the western region of the country. Understanding the significance of FGM from the medical, psychological, and social perspectives might spot the light on clear and specific legislation preventing the practice of FGM in Saudi Arabia. To do so, nationwide studies regarding the practice of FGM need to be conducted in Saudi Arabia to determine the prevalence and actual impact of FGM at the national and subnational levels, as there are limited studies addressing this issue.
